# Adaptive divergence and underlying mechanisms in response to salinity gradients between two *Crassostrea* oysters revealed by phenotypic and transcriptomic analyses

**DOI:** 10.1111/eva.13370

**Published:** 2022-04-18

**Authors:** Ziyan Zhang, Ao Li, Zhicai She, Xuegang Wang, Zhen Jia, Wei Wang, Guofan Zhang, Li Li

**Affiliations:** ^1^ CAS and Shandong Province Key Laboratory of Experimental Marine Biology, Center for Ocean Mega‐Science, Institute of Oceanology Chinese Academy of Sciences Qingdao China; ^2^ University of Chinese Academy of Sciences Beijing China; ^3^ Laboratory for Marine Biology and Biotechnology Pilot National Laboratory for Marine Science and Technology Qingdao China; ^4^ Laboratory for Marine Fisheries Science and Food Production Processes Pilot National Laboratory for Marine Science and Technology Qingdao China; ^5^ National and Local Joint Engineering Key Laboratory of Ecological Mariculture, Institute of Oceanology Chinese Academy of Sciences Qingdao China; ^6^ Guangxi Key Laboratory of Beibu Gulf Marine Biodiversity Conservation, College of Marine Sciences Beibu Gulf University Qinzhou China

**Keywords:** adaptive divergence, oysters, phenotypic and transcriptomic analyses, related species, salinity gradient

## Abstract

Comparing the responses of closely related species to environmental changes is an efficient method to explore adaptive divergence, for a better understanding of the adaptive evolution of marine species under rapidly changing climates. Oysters are keystone species thrive in intertidal and estuarine areas where frequent environmental disturbance occurs including fluctuant salinity. The evolutionary divergence of two sister species of sympatric estuarine oysters, *Crassostrea hongkongensis* and *Crassostrea ariakensis*, in response to euryhaline habitats on phenotypes and gene expression, and the relative contribution of species effect, environment effect, and their interaction to the divergence were explored. After a 2‐month outplanting at high‐ and low‐salinity locations in the same estuary, the high growth rate, percent survival, and high tolerance indicated by physiological parameters suggested that the fitness of *C. ariakensis* was higher under high‐salinity conditions and that of *C. hongkongensis* was higher under low‐salinity conditions. Moreover, a transcriptomic analysis showed the two species exhibited differentiated transcriptional expression in high‐ and low‐salinity habitats, largely caused by the species effect. Several of the important pathways enriched in divergent genes between species were also salinity‐responsive pathways. Specifically, the pyruvate and taurine metabolism pathway and several solute carriers may contribute to the hyperosmotic adaptation of *C. ariakensis*, and some solute carriers may contribute to the hypoosmotic adaptation of *C. hongkongensis*. Our findings provide insights into the phenotypic and molecular mechanisms underlying salinity adaptation in marine mollusks, which will facilitate the assessment of the adaptive capacity of marine species in the context of climate change and will also provide practical information for marine resource conservation and aquaculture.

## 1 INTRODUCTION

Numerous studies have reported acceleration in the global hydrological cycle since 1960, resulting in an increase in salinity in high‐salinity areas and a decrease in salinity in low‐salinity areas (Cheng et al., 2020). These changes in climate may expose organisms to conditions that are not optimum or outside their primary survival range. Therefore, it is essential to investigate the salinity adaptation of populations in response to environmental changes. Estuaries naturally form various salinity gradients along the coastline, particularly during rainy seasons, and are highly susceptible to anthropogenic activities and climate change effects. Thus, estuarine organisms are subject to rapid fluctuations in various physical parameters, such as salinity, temperature, pH, and dissolved oxygen.

Populations can respond to climate change through two essential strategies: adaptive evolution and phenotypic plasticity. The adaptability of populations to changing environments depends on the species and environment and can be further classified into the species effect, environment effect (nongenetic plastic responsiveness to environmental change), and species–environment interaction. Many studies have been conducted to determine the species (genetic) or environment effects by common garden (Pratlong et al., 2015; Vesakoski et al., 2009; Wang, Weinberger, et al., 2017) and environmental simulation experiments (Evans et al., 2017; Liu et al., 2019). However, limited studies have quantified the relative contribution of the species effect, environment effect, and their interaction in the molecular response of organisms to changing environments (Eierman & Hare, 2016; Oostra et al., 2018).

Comparison of the various responses of related species to environmental gradients is an efficient method to explore the adaptive divergence of species to climate change. Different adaptive performances are represented by divergence in phenotypic and molecular responses between intraspecies populations and related species along environmental gradients. Fitness‐related traits, including growth, survival, and reproduction (Conover et al., 2006; Robinson, 2013; Sanford & Kelly, 2011; Sherman & Ayre, 2008; Vesakoski et al., 2009); physiological activities (Brennan et al., 2018; Li et al., 2017); behaviors (Bingham & Young, 1991); genetic structure (Andre et al., 2011; Chen et al., 2021; Jones et al., 2012; Li et al., 2006; Popovic & Riginos, 2020; Zhan et al., 2009); and comparative transcriptomic analyses within species and among related species (Bernal et al., 2020; Brennan et al., 2015; Kitano et al., 2019; Li et al., 2018; Popovic & Riginos, 2020; Porcelli et al., 2015; Raeymaekers et al., 2017; Wang et al., 2020; Yuan et al., 2017; Zhu et al., 2020) have been widely used to evaluate the adaptive responses of marine and aquatic organisms to environmental gradients such as temperature, salinity, and altitude. A comparative transcriptomic analysis can detect microscopic variation in gene expression and quantify the species effect, environment effect, and species–environment interaction.

The oyster, a keystone species of sessile bivalves living in salinity‐fluctuating estuaries and intertidal zones, is an excellent model system for studying the response of marine organisms to climate change. *Crassostrea ariakensis* and *Crassostrea hongkongensis* are sister species adapted to living in estuaries based on phylogenetic analysis of mitochondrial and genomic DNA and Kimura 2‐parameter genetic distances (Ren et al., 2010; Wang et al., 2004; Xiao et al., 2018; Yu & Li, 2012; Zhang et al., 2022). *C. ariakensis* is discontinuously distributed along the coastal estuaries of both northern and southern China, in addition to other areas of East Asia (Wang et al., 2004). In contrast to its previous widespread distribution, *C. ariakensis* is now sporadically distributed in northern China due to a steady decline in the abundance of natural resources in recent decades. *C. hongkongensis* is only distributed in southern China, predominately in Fujian, Guangdong, and Guangxi Provinces, where ecological niches of the two species overlap. Moreover, *C. hongkongensis*, which is a major aquaculture species in China with an annual output of 179.84 × 10^6^ t in 2019 (Fisheries & Fisheries Administration of the Ministry of Agriculture & Rural Affairs, 2020), is prone to hypersalinity‐related mass mortality according to local fishermen, which threatens the oyster industry in southern China. Consequently, local fishermen have partly shifted toward farming *C. ariakensis*, which do not suffer from salinity‐related mass mortality. Besides, several studies have explored the hybridization of *C. hongkongensis* with *C. ariakensis* for better performance in growth and resistance of the former (Qin et al., 2020, 2021).

Although previous transcriptomic analysis‐based studies have reported the molecular responses to osmotic changes under various salinities in *Crassostrea* oysters (Liu et al., 2019; Meng et al., 2013; Xiao et al., 2018; Yan et al., 2017), the fine‐scale evolutionary divergence between these two closely related species to salinity changes remains unknown as does the relative contribution of the species effect, environment effect, and the interaction between species and environment to adaptive divergence. In this study, we conducted a two‐factor comparative experiment in an estuary with salinity gradients in which the growth, survival, and resistance‐ and metabolism‐related physiological responses of *C*. *ariakensis* and *C. hongkongensis* were determined. In addition, a transcriptomic analysis was performed to characterize the relative contribution of species and environment factors to the divergent molecular responses of the two oyster species to different salinity gradients, and to reveal the mechanisms underlying the adaptive divergence between two species. The dissection of the fine‐scale divergence in salinity adaptation between the two sister species will not only lay the theoretical foundation for the appropriate culture conditions and genetic improvement in conservation and aquaculture of the oyster, but it will also provide new insights into the adaptive evolution of mollusks and other invertebrates.

## 2 MATERIALS AND METHODS

### 2.1 Sample preparation and monitoring of seawater salinity and temperature

**FIGURE 1:**
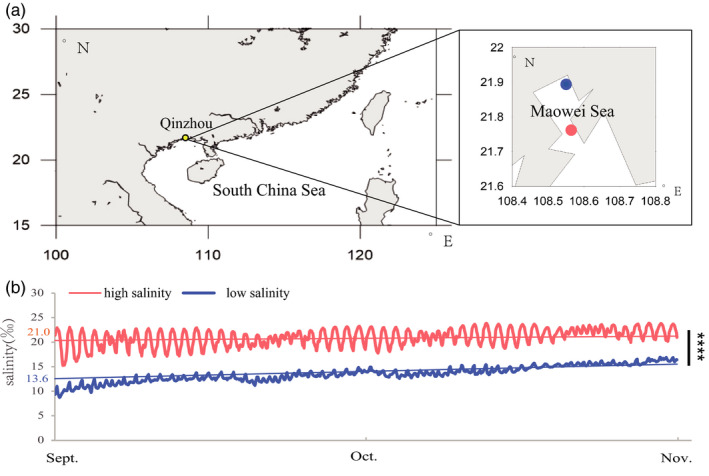
Sampling locations and seawater salinity. (a) Map showing the sampling locations of *Crassostrea ariakensis* and *C. hongkongensis* cultivation in estuaries along the coasts of Qinzhou, Guangxi Province, in China. Yellow spot indicates the cultivation estuary Maowei Sea. Blue spot indicates low‐salinity environment, and light red spot indicates high‐salinity environment. (b) Seawater salinity of the two sampling locations recorded by a conductivity logger at 1‐h intervals. Light red and blue numbers on the left separately indicate the average salinity of the high‐salinity and low‐salinity environments, respectively, during 2 months of acclimatization (September to November 2019). Asterisks indicate a significant difference (****p < 0.0001)

Naturally wild *C. ariakensis* and *C. hongkongensis* were collected from the same coastal estuary in Qinzhou, Guangxi Province (21.9°N, 108.5°E), in southern China. The collection site had a water salinity of 21 ± 1.7‰ and a water temperature of 20 ± 2.7°C. For broodstock conditioning, individuals of two species were acclimatized to sexual maturity in a hatchery of our institute in Qinzhou under the same conditions for a month, where the salinity was controlled as 20 ± 1.5‰ and the temperature was gradually increased from 18°C to 26°C. Afterward, 30 × 30 breeding patterns were conducted for each species. After identifying parent males and females, the eggs of 30 female parents were dissected, mixed, and divided into 30 beakers, and artificial insemination was conducted simultaneously for each species with the sperm from each of the 30 male parents in June 2019. The larvae of the two species were cultured in the hatchery under the same salinity and temperature conditions controlled as 20 ± 1.5‰ and 24 ± 1.0°C, respectively, until they attached to the scallop shell substrates two weeks postfertilization. When the mean size of settled spats for both species reached 5 mm, the scallop shell substrates were connected on hanging ropes under sea surface at two sites with a relatively high (approximate range: 18–27‰, average: 22.5‰) or low (approximate range: 6–20‰, average: 14.5‰) salinity in the Maowei Sea in September 2019 (Figure 1a). The four experimental groups comprised *C. ariakensis* (AR) and *C. hongkongensis* (HK) oysters acclimatized to high‐salinity (HS) or low‐salinity (LS) environments. After two months of cultivation until November 2019, gill tissues were sampled in situ from each of the four experimental groups (*n* = 20, five biological replicates per group). These samples were immediately frozen in liquid nitrogen and preserved in a −80°C freezer for physiological determination and RNA extraction.

Seawater salinity and temperature records of the two field habitats during acclimatization (September to November) were monitored using HOBO Conductivity U24 Data Loggers (ONSET) with a time interval of 1 h. These devices were placed at the same depth as the cultured oysters.

### 2.2 Measurement of growth and survival in the wild

The growth rates (based on shell height) and percent survival (based on number of survivors) of oysters in each experimental group were measured at the beginning (September 2019) and end (November 2019) of cultivation in the Maowei Sea. Because there were no significant differences in the percent survival after short‐term (2 months) acclimatization among the four experimental groups, we compared the percent survival between the two species after long‐term (9 months) acclimatization in June 2020.

### 2.3 Physiological measurement

Physiological parameters related to resistance upon oxidative damage, such as superoxide dismutase (SOD), malondialdehyde (MDA), and metabolic enzyme activities, including pyruvate kinase (PK), phosphoenolpyruvate carboxykinase (PEPCK), and citrate synthase (CS), in 20 individuals (five individuals per species at each site) were determined to explore the physiological divergence in the two species exposed to different salinity gradients. Approximately 0.02 g of frozen gill tissue was obtained from each sample, mixed with precooled 0.9% physiological saline or other buffer solutions in a weight‐to‐volume ratio of 1:9, and ground to 10% tissue suspension using an electric homogenizer on ice. The tissue suspension was centrifuged at 700 × *g* for 10 min at 4°C in a Multifuge X1R Centrifuge (Thermo Fisher Scientific), and the supernatant was diluted with physiological saline or buffer solution to an appropriate concentration for analysis.

Before determining each physiological substance, proteins were determined using a total protein assay kit (Nanjing Jiancheng Bioengineering Institute) with bicinchoninic acid following the colorimetry instructions for the subsequent calculations of other target enzyme activities. The SOD activity was determined using a total SOD assay kit (Beyotime) with nitroblue tetrazolium. We determined the content of MDA using an MDA assay kit (Nanjing Jiancheng Bioengineering Institute) with thiobarbituric acid as the substrate. The activities of PK, PEPCK, and CS were determined separately using corresponding kits from the Nanjing Jiancheng Bioengineering Institute. The assays of all these enzymes and metabolites were conducted following the instructions of the kit manufacturers, and the optical density values of each reaction product were measured using a Varioskan Flash Multimode Reader (Thermo Fisher Scientific) for further calculating the activity of each enzyme and the metabolite content.

### 2.4 RNA extraction

To understand the divergent transcriptional responses of the two *Crassostrea* species to different salinity conditions, 20 gill transcriptomes (five for each species at each site) were sequenced from the same individuals used for the physiological measurements above. Total RNA was extracted from approximately 0.02 g of frozen gill tissue using an RNAprep Pure Tissue Kit (Tiangen) according to the manufacturer's instructions (*n* = 20). The RNA integrity and concentrations were assessed using a DYY‐12 electrophoresis apparatus (Liuyi) with 1.0% gel and a NanoDrop 2000 spectrophotometer (Thermo Fisher Scientific) at 260 nm.

### 2.5 cDNA library construction and sequencing

The cDNA library of the RNA samples was constructed using the Illumina TruSeq™ RNA Sample Preparation Kit (Illumina). Then, the cDNA libraries were sequenced using an Illumina HiSeq X Ten platform (Illumina) based on sequencing by synthesis technology with the PE150 strategy (meaning paired‐end sequencing and 150 bases for each read), thereby yielding raw data. Raw data were filtered to obtain high‐quality clean data according to the following protocol: (1) removing the adapter sequence, (2) ensuring that the N base content remained below 10%, and (3) removing bad‐quality data (over 50% of the bases of the entire read recorded quality values Q ≤ 5).

This study used the existing genomes of *C. ariakensis* (BioProject Accession: PRJNA715058 at NCBI; Li et al., 2021) and *C. hongkongensis* (BioProject Accession: PRJNA592306 at NCBI; Zhang et al., 2022) as references for the corresponding species to complete sequence mapping and subsequent analysis. The reads were mapped using HISAT2 (Kim et al., 2015) with parameters “‐dta ‐p 6 ‐max ‐intronlen 5000000,” following which the mapped reads were assembled using StringTie (Pertea et al., 2015).

### 2.6 Identification of single nucleotide polymorphism sites

Based on the mapping results obtained using HISAT2, the HaplotypeCaller tool with parameters “‐dontUseSoftClippedBases ‐stand_call_conf 20.0 ‐stand_emit_conf 20.0” in GATK (McKenna et al., 2010) was used to identify single‐base mismatches between the sample sequence and reference genome to identify single nucleotide polymorphism (SNP) sites. SNPs were filtered according to the following standards: (1) ensuring no more than three consecutive single‐base mismatches in the range of 35 bp and (2) ensuring that the recorded quality values of the SNP exceeded 2.0 (QD ≥ 2.0). These SNPs were annotated using SnpEff (Cingolani et al., 2012).

### 2.7 Filtering of orthologous genes

To compare the transcriptional responses to salinity between *C. ariakensis* and *C. hongkongensis*, the protein sequences predicted by TransDecoder v5.5.0 (Haas & Papanicolaou, 2013) were grouped into clusters of orthologous genes, excluding paralogs, using OrthoFinderv2.2.7 (Emms & Kelly, 2015) with default parameters. We removed orthologs if the e‐value of the sequence similarity alignment was larger than 1e^−5^ to improve prediction accuracy. This assessment resulted in 16,130 pairs of one‐to‐one orthologous genes.

### 2.8 Quantification and annotation of filtered orthologous genes

Orthologous genes were quantified by mapped reads against the reference genomes using StringTie (Pertea et al., 2015) and were annotated according to following databases: Gene Ontology (GO, http://www.geneontology.org/), Kyoto Encyclopedia of Genes and Genomes (KEGG, http://www.genome.jp/kegg/), and NCBI nonredundant protein sequences (NR, ftp://ftp.ncbi.nih.gov/blast/db/).

To reasonably measure gene expression, fragments per kilobase of transcript per million fragments mapped (FPKM) was used as an index to normalize the number of mapped reads with the length of the transcript. The following calculation formula was applied:

$$\begin{array}{c} \text{FPKM}=\\ \left(\text{cDNA}\;\text{fragments}/\left[\text{mapped}\;\text{fragments}\;(\text{millions})\times \text{transcript}\;\text{length}\;\text{(kb)}\right]\right) \end{array}$$

where cDNA fragments indicate the number of fragments (i.e., the paired‐end reads, same as below) mapped to a gene, and mapped fragments (millions) represent the total number of fragments mapped to all genes (10^6^ bases). The unit of the transcript length (kb) was 10^3^ bases.

### 2.9 Differential gene expression analyses

Based on the results of the orthologous genes, we performed a likelihood‐ratio test on the expression data using LPESeq (Gim et al., 2016) and ensured that the q‐value was less than 0.01. Genes with |log_2_ (fold change)| ≥ 1 and q‐value <0.01 were classified as differentially expressed genes (DEGs).

Differential gene expression analyses were performed, which resulted in several differential expression gene sets that were categorized into three effects: (1) species effect (AR_HS vs. HK_HS and AR_LS vs. HK_LS), which causes differential expression between two species in the same salinity environment; (2) environment effect (AR_HS vs. AR_LS and HK_HS vs. HK_LS), which leads to differential expression in the same species exposed to different salinity environments; (3) species–environment interaction (AR_HS vs. HK_LS and AR_LS vs. HK_HS), which causes differential expression between the two species under different salinity conditions.

### 2.10 Functional enrichment analyses

Functional enrichment analyses were performed for the gene sets of interest. Enrichment of Gene Ontology (GO) categories for each pairwise comparison was performed with GOseq (Young et al., 2010) R packages based on the Wallenius' noncentral hypergeometric distribution with *p* < 0.05, and topGO (Alexa & Rahnenfuhrer, 2010) with R (firstSigNodes = 10). The resulting GO terms were divided into biological processes, cellular components, and metabolic functions, according to their function. To understand the relevance of functional pathways, we annotated the DEG sets according to the Kyoto Encyclopedia of Genes and Genomes (KEGG) pathway database (Kanehisa et al., 2008), and KEGG pathway enrichment analyses were conducted to analyze the biological functions of particular gene sets using KOBAS (Mao et al., 2005).

### 2.11 Weighted gene correlation network analysis

A weighted gene correlation network analysis (WGCNA) was performed using the expression data (FPKM) of orthologous genes and physiological response trait data of all the biological replicates of the two species exposed to two salinity gradients. The weighted gene correlation network was constructed using the R package WGCNA (Langfelder & Horvath, 2008) to identify modules of co‐expressed genes associated with each species, salinity gradients, and measured physiological responses (parameters = blockwiseModules; power = 4; mergeCutHeight = 0.25; min ModuleSize = 30; TOMType = unsigned). The traits of each biological replicate were as follows: (i) continuous variables of the activity or content of SOD, MDA, PK, PEPCK, and CS; and (ii) discrete variables of 0 or 1 for species of *C. ariakensis* or *C. hongkongensis* and for high or low salinity. After filtering out the low‐expression genes (mean FPKM < 1) to improve the accuracy of the resulting network, correlation networks were constructed based on pairwise gene expression correlations across all the samples. The modules were defined as highly interconnected clusters, and genes within the same modules were highly correlated. To determine biologically significant modules, module eigengenes were used to calculate the correlation coefficients with samples and sample traits.

The specific gene modules associated with species, salinity, resistance, and metabolism were investigated. For genes in the modules of interest, GO and KEGG pathway enrichment analyses were conducted to analyze the major biological functions of the modules. For the enrichment analyses, a list of genes in each module was compared with the background genes, which were the total orthologous genes we predicted (16,130 for each species). The significantly enriched GO terms and pathways were defined by a hypergeometric test with *p* < 0.05.

### 2.12 Statistical analysis

The normal distribution and homogeneity of the variances were tested using the Shapiro–Wilk and *F* tests, respectively. Moreover, with GraphPad Prism 8, statistical significance in salinity and temperature between the two sampling sites was tested by Welch's *t*‐test, and that in shell height, percent survival, and each physiological index of the four experimental groups was tested by two‐way ANOVA with post hoc analyses. PCA was performed based on the FPKM of the total orthologous genes with R. Iqtree (Minh et al., 2020) was used to construct a phylogenetic tree based on SNPs. Venn diagrams of gene sets responding to species effect, environment effect, and species–environment interaction were created using the online tool jvenn (Bardou et al., 2014), and heatmaps of DEGs were constructed using the pheatmap R package (Kolde, 2019). Moreover, the relative importance of the species effect, environment effect, and the interaction between species and environment to differential expression was evaluated via |log_2_(fold change)| of each orthologous gene using the Mann‐Whitney *U* test in SPSS v. 21.0 (SPSS Inc.).

## 3 RESULTS

### 3.1 Salinity and temperature of the two acclimatized habitats

We observed significant salinity differences between the two culture sites during the 2‐month acclimatization period (*p* < 0.0001, Figure 1b, Table S1), and the difference in the average salinity (HS: 21.0 ± 1.7‰; LS: 13.6 ± 1.6‰) between the two habitats was 7.4‰. In addition, the overall magnitude of salinity fluctuation at the two sites was similar during the experiment (HS: 8.6‰; LS: 8.2‰), although the daily fluctuation in the high‐salinity habitat exceeded that in the low‐sanity habitat. Meanwhile, the mean temperature differences between the two sampling sites were less than 1°C (Figure S1).

### 3.2 Growth and survival of the oyster species

**FIGURE 2:**
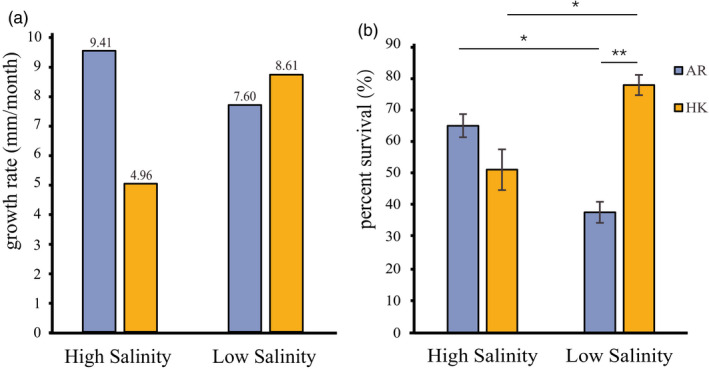
Fitness‐related traits of *Crassostrea ariakensis* and *C. hongkongensis* acclimatized to two salinity gradients. (a) The growth rates based on the shell heights of *C. ariakensis* and *C. hongkongensis* acclimatized to high‐ and low‐salinity environments from September to November 2019. (b) The percent survival of *C. ariakensis* and *C. hongkongensis* after long‐term (September 2019 to June 2020) acclimatization to high‐ and low‐salinity environments due to the lack of significant differences after short‐term acclimatization (September to November 2019). Asterisks indicate significant differences (*p < 0.05, **p < 0.01). Error bars indicate SD. AR: *C. ariakensis*, HK: *C. hongkongensis*

After the 2‐month acclimatization period in different salinity gradients, the shell height of *C. hongkongensis* in the low‐salinity environment was significantly higher than that in the high‐salinity environment (*p* < 0.0001, Figure S2, Table S2). Moreover, the population‐level growth rate of *C. hongkongensis* was higher in the low‐salinity environment (8.61 mm/month) than in the high‐salinity environment (4.96 mm/month), whereas for *C. ariakensis*, the population‐level growth rate was higher in the high‐salinity environment than in the low‐salinity environment (high salinity: 9.41 mm/month, low salinity: 7.60 mm/month; Figure 2a, Figure S2). Consistent with the growth rate, the percent survival of *C. hongkongensis* was significantly higher in the low‐salinity environment than in the high‐salinity environment, whereas the percent survival of *C. ariakensis* was higher in the high‐salinity environment than in the low‐salinity environment (*p* < 0.05; Figure 2b, Table S2).

### 3.3 Physiological response variations

**FIGURE 3:**
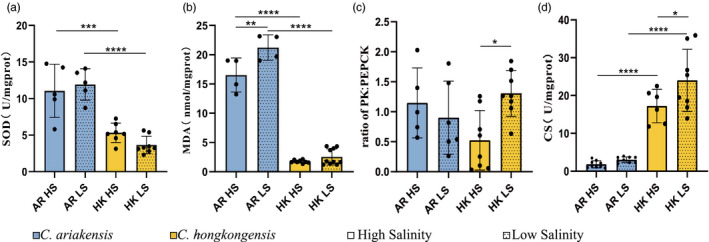
Physiological indicators of *Crassostrea ariakensis* and *C. hongkongensis* acclimatized to two salinity gradients. Physiological indicators of the two oyster species after 2 months of acclimatization (September to November 2019) in high‐ and low‐salinity environments, including the superoxide dismutase activity (SOD, a), malondialdehyde content (MDA, b), ratio of pyruvate kinase to phosphoenolpyruvate carboxykinase (PK:PEPCK, c), and citrate synthase activity (CS, d). Asterisks indicate significant differences (*p < 0.05, **p < 0.01, ***p < 0.001, and ****p < 0.0001). Error bars indicate SD. Spots indicate the measurement results of each sample. AR: *C. ariakensis*, HK: *C. hongkongensis*; HS, high salinity; LS, low salinity

Two months postoutplanting in different salinity gradients for the two species, significant physiological divergences between *C. ariakensis* and *C. hongkongensis* were observed, the significance tests of which are listed in Table S2. For the antioxidant process, the SOD activity of *C. hongkongensis* was slightly higher in oysters in the high‐salinity environment than in those in the low‐salinity environment, whereas the SOD activity of *C. ariakensis* was slightly higher in low‐salinity conditions than that in high‐salinity conditions (Figure 3a). In addition, the MDA content was significantly lower under high‐salinity conditions than under low‐salinity conditions in *C. ariakensis* (*p* < 0.01, Figure 3b). For the metabolic enzymes, the activity ratio of PK:PEPCK was significantly lower for *C. hongkongensis* under high‐salinity conditions than under low‐salinity conditions (*p* < 0.05, Figure 3c), whereas that of *C. ariakensis* showed a downward trend under low‐salinity conditions in contrast to that under high‐salinity conditions. Moreover, the CS was more active in the low‐salinity gradient than in high‐salinity gradient for *C. hongkongensis* (*p* < 0.05, Figure 3d), and the same trend was observed for *C. ariakensis*.

### 3.4 Summary of RNA‐seq for the two *Crassostrea* species

A total of 63.07 GB of clean data was obtained from all *C. ariakensis* samples, and the Q30 of the clean data exceeded 95.0%. Moreover, when mapping clean reads to the *C. ariakensis* reference genome, the mapped reads were 78.95% on average for the *C. ariakensis* samples. A total of 87.70 GB of clean data for *C. hongkongensis* samples with a Q30 > 93.7% was mapped above 80.69% to the *C. hongkongensis* genome (Table S3). Moreover, 34,729 and 40,714 genes were identified, respectively, in *C. ariakensis* and *C. hongkongensis*, of which 32,723 and 38,021 were annotated. To improve the comparability of the gene expression between the different species, we identified a total of 16,130 pairs of one‐to‐one orthologous genes in *C. ariakensis* and *C. hongkongensis* for differential expression analysis and WGCNA, of which 15,945 pairs of orthologous genes were annotated.

### 3.5 Clustering of biological samples based on SNPs and gene expression

**FIGURE 4:**
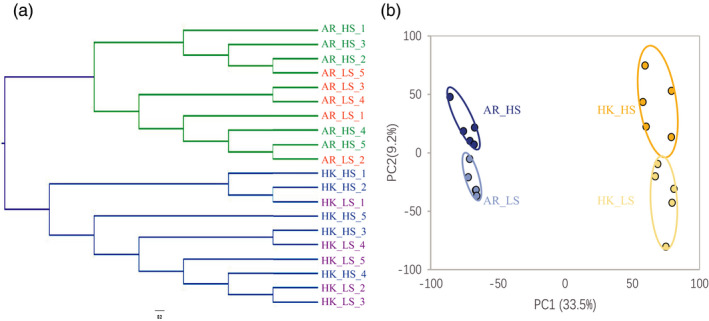
Single nucleotide polymorphism (SNP) phylogenetic tree and principal component analysis (PCA) plots. (a) Clustering based on the SNP of *Crassostrea ariakensis* and *C. hongkongensis* cultured simultaneously in low‐ and high‐salinity environments for 2 months. AR_HS_1–AR_HS_5: five samples of *C. ariakensis* cultured in a high‐salinity environment; AR_LS_1–AR_LS_5: five samples of *C. ariakensis* cultured in a low‐salinity environment; HK_HS_1–HK_HS_5: five samples of *C. hongkongensis* cultured in a high‐salinity environment; and HK_LS_1–HK_LS_5: five samples of *C. hongkongensis* cultured in a low‐salinity environment. (b) PCA. The distribution of spots and circles indicates the clustering and variance based on the FPKM of all the orthologous genes in low‐ and high‐salinity environments for *C. ariakensis* and *C. hongkongensis*. The variance proportions are shown on the coordinate axis. Each spot indicates one biological sample. Blue and yellow shapes indicate *C. ariakensis* and *C. hongkongensis*, respectively. Dark and light colors indicate high salinity and low salinity, respectively

Based on the SNP phylogenetic tree, oysters of the same species dwelling in the two salinity environments mixed into one branch, and all biological samples were clustered into two major branches: *C. ariakensis* and *C. hongkongensis* (Figure 4a). According to the PCA, PC1, which accounted for 33.5% of the total variance, separated the gene expression patterns between the two species. PC2, which accounted for 9.2% of the total transcriptional variance, separated the samples cultured in different salinity gradients for both species (Figure 4b).

### 3.6 Identification of DEGs for species effect, environment effect, and species–environment interaction

**FIGURE 5:**
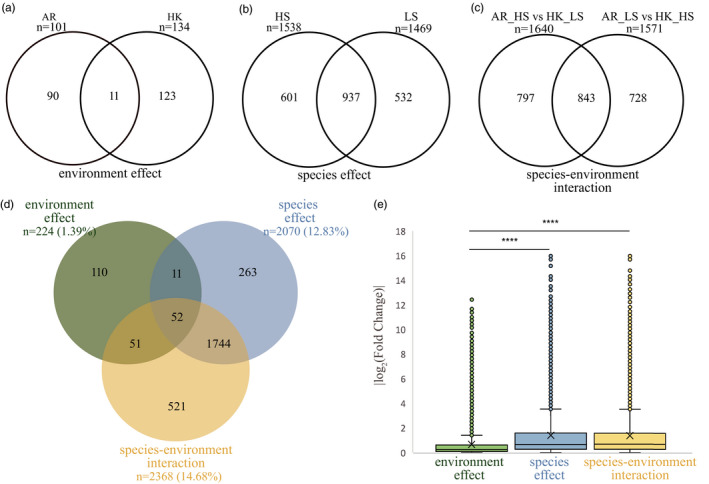
Venn diagrams and expression variances of environment effect, species effect, and species–environment interaction. Venn diagrams of the number of differentially expressed genes (DEGs): (a) between the salinity gradients for *Crassostrea ariakensis* and *C*. *hongkongensis* denoted as the environment effect; (b) between the two species in high‐ and low‐salinity environments denoted as the species effect; (c) affected by the interaction between species and environment, denoted as the species–environment interaction; and (d) the union of DEGs affected by these three factors. The proportions indicate the number of DEGs separately affected by each factor in contrast to that of all the orthologous genes. Overlaps indicate genes differentially expressed in response to multiple factors. AR: *C. ariakensis*, HK: *C. hongkongensis*, LS: low salinity, HS: high salinity. (e) Boxplots of |log_2_(fold change)| of all the orthologous genes in two species, representing the level of differential expression by each factor. The little circles represent the outlier |log_2_(fold change)| values for genes showing each of the three effects. The five horizontal lines of the box represent the positions of the maximum, third quartile (Q3), median (Q2), first quartile (Q1), and minimum, respectively. “X” on each boxplot indicates the mean of the |log_2_(fold change)|, which was 0.66 for environment effect, 1.39 for species effect, and 1.37 for species–environment interaction. The SD of the |log_2_(fold change)| for environment effect, species effect, and species–environment interaction was 1.22, 1.90, and 1.84, respectively. Asterisks indicate significant differences (****p < 0.0001)

In total, 2752 genes (17.06%) of 16,130 orthologous genes were significant DEGs through a differential expression analysis (|log_2_(fold change)| ≥ 1, q‐value < 0.01). In the differential expression analysis, 101 genes from AR_HS vs. AR_LS and 134 genes from HK_HS vs. HK_LS showing the environment effect were differentiated between the high‐salinity and low‐salinity environments, of which only 11 DEGs overlapped (Table S4, Figure 5a). More differential genes were found between the two species at high salinity and low salinity: AR_HS vs. HK_HS (1538) and AR_LS vs. HK_LS (1469) (Table S4, Figure 5b). Similarly, DEGs affected by species–environment interaction were observed from AR_HS vs. HK_LS (1640) and AR_LS vs. HK_HS (1571) (Table S4, Figure 5c).

The DEGs affected by species differences, environment, and their interaction are shown in the Venn diagram in Figure 5d. Among the 224 DEGs showing the environment effect, only 110 were DEGs for the environment factor alone. Moreover, of the 2070 DEGs showing significant differences between the species, 263 only showed the species effect, and 521 of 2368 DEGs were only affected by the species–environment interaction. A large number of DEGs (1744 of the total 2752) were affected by both the species and the species–environment interaction (Figure 5d). Across all orthologous genes, the logarithm of absolute expression change (|log_2_(fold change)|) between the different salinity gradients was significantly smaller than that affected by the species effect and species–environment interaction (*p* < 0.0001, Figure 5e, Table S1). Only 16.14% of all the transcripts showed more than a twofold change in expression between the salinity gradients than the 36.75% and 37.40% affected by the species effect and species–environment interaction, respectively.

*Crassostrea hongkongensis* showed a higher expressional plasticity between two salinity gradients according to the fold change of DEGs affected by the environment (*p* < 0.01, Figure S3a, Table S1). Similarly, among the DEGs between species, the expressional difference between the two salinity gradients was also greater in *C. hongkongensis* (*p* < 0.05, Figure S3b, Table S1).

### 3.7 Gene modules correlated with species, environment, and physiological parameters derived from WGCNA

**FIGURE 6:**
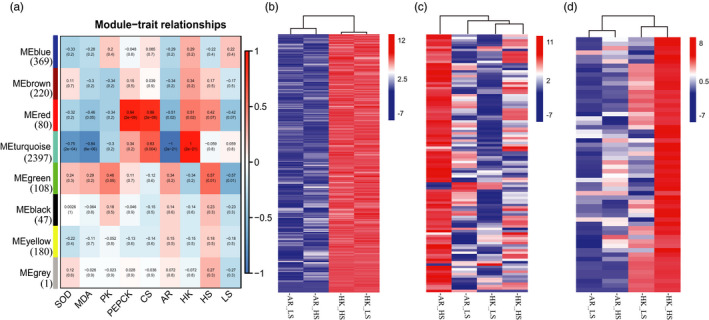
Weighted gene correlation network analysis (WGCNA) of orthologous genes in *Crassostrea ariakensis* and *C. hongkongensis* at two salinity gradients. (a) Module–samples/traits relationships. Each row corresponds to one module, labeled with ME (module eigengene), and one color. The number of genes in each module is indicated on the left. The color of the boxes represents −log(*p*), the Fisher exact test *p*‐value, according to the color legend on the right. The numbers in the boxes indicate the *p*‐value and correlation between each module and trait. (b–d) Heatmaps of the expression of genes included in the (b) species‐ and resistance‐related turquoise module, (c) salinity‐related green module, and (d) species‐ and metabolism‐related red module. Red boxes indicate high expressions, and blue boxes indicate low expressions. AR: *C. ariakensis*; HK: *C. hongkongensis*; HS, high salinity; LS, low salinity

All the orthologous genes of the 20 samples were classified into eight gene co‐expression modules according to the expression pattern correlation through WGCNA (Figure S4). These modules were then used to conduct a correlation analysis with SOD, MDA, PK, PEPCK, CS, and various species (AR\HK) and salinity (HS\LS) classifications. The analysis resulted in the following three key modules that were highly correlated with multiple traits (*p* < 0.05, Figure 6a). Among all the module genes, more than 70% (2477 out of 3402) showed the species effect.

The turquoise module (*n* = 2397), which was related to physiological parameters that indicate resistance upon oxidative damage, such as SOD (*ρ* = −0.75, *p* = 2e−04) and MDA (*ρ* = −0.84, *p* = 6e−06), showed a significant negative correlation with *C. ariakensis* (*ρ* = −1, *p* = 2e−21) but was positively correlated with *C. hongkongensis* (*ρ* = 1, *p* = 2e−21). Genes in the turquoise module exhibited divergent expression between two species and were highly expressed in *C. hongkongensis* in contrast to *C. ariakensis* (Figure 6b).

The green module (*n* = 108), a key module related to salinity response, showed a significant correlation with high‐salinity (*ρ* = 0.57, *p* = 0.01) and low‐salinity (*ρ* = −0.57, *p* = 0.01) environments. The expression of 108 genes in the green module was upregulated under high‐salinity conditions in contrast to low‐salinity conditions and was higher in *C. ariakensis* than in *C. hongkongensis* (Figure 6c).

The red module (*n* = 80), which was related to the key enzymes in respiration metabolism processes, such as PEPCK (*ρ* = 0.94, *p* = 2e−09) and CS (*ρ* = 0.86, *p* = 2e−06), was another species‐correlated module showing a negative correlation with *C. ariakensis* and a positive correlation with *C. hongkongensis* (AR: *ρ* = −0.51, *p* = 0.02, HK: *ρ* = 0.51, *p* = 0.02). Moreover, the eigengenes of the red module were highly expressed in the high‐salinity environment in *C. hongkongensis* than in other scenarios (Figure 6d).

### 3.8 Functional enrichment analyses of species divergent genes and salinity‐responsive genes

We conducted functional enrichment analyses of the species divergent genes (DEGs between species and genes in species‐correlated modules) and salinity‐responsive genes (DEGs between salinity gradients and genes in salinity‐correlated modules) to identify the significant pathways responding to salinity gradients and species divergence. Unique and common pathways were enriched for the species divergent genes and salinity‐responsive genes. All significantly enriched GO terms or KEGG pathways with their IDs and *p*‐values are shown in File S1 (*p* < 0.05), and detailed information of the enriched DEGs in File S1 is provided in File S2.

The functional responses to different salinity gradients showed differences between the two oyster species in terms of GO and KEGG enrichment (*p* < 0.05). A total of 101 salinity‐responsive DEGs in *C. ariakensis* were enriched in 45 GO terms and five KEGG pathways (*p* < 0.05, File S1). The pyruvate metabolic and carnosine biosynthetic processes, the beta‐alanine metabolism, protein processing in endoplasmic reticulum, and sphingolipid metabolism were uniquely enriched. In contrast, 134 salinity‐responsive DEGs in *C. hongkongensis* were enriched in 54 GO terms and eight KEGG pathways (*p* < 0.05, File S1), among which the taurine biosynthetic process and transcription factor activity, ABC transporters, several carbohydrate metabolism pathways and FoxO signaling pathway showed unique enrichment. The arginine catabolic process, transmembrane transport, and free amino acid (FAA) metabolism, including arginine, proline, and histidine, were enriched in environment‐responsive DEG sets for both species (File S1).

Genes in the salinity‐correlated green module were enriched in eight KEGG pathways: FAA metabolism (such as arginine, proline, and beta‐alanine), pyruvate metabolism, FoxO signaling pathways, citrate cycle (TCA cycle), glycolysis/gluconeogenesis, neuroactive ligand–receptor interaction, and CYP450, in addition to 39 GO terms, including the pyruvate metabolic process, taurine biosynthetic and metabolic processes, transmembrane transporter activity, and arginine catabolic process (File S1, *p* < 0.05).

Differentially expressed genes between species were enriched in unique and common pathways in the high‐ and low‐salinity environments (*p* < 0.05). Of the 1538 DEGs in the high‐salinity environment, 62 GO terms and five KEGG pathways were enriched, with unique enrichment in transcription factor activity and the proteasome (*p* < 0.05, File S1). Meanwhile, of the 1469 DEGs in the low‐salinity environment, 52 GO terms and seven KEGG pathways were enriched, of which the glutamine family amino acid biosynthetic process, cytochrome P450, the regulation of autophagy, and ribosomes were uniquely enriched (*p* < 0.05, File S1). The enrichment results of both DEG sets for the species effect in the high‐ and low‐salinity environments included numerous transporters in terms related to transporter activity (such as 20 solute carrier [SLC] subfamilies, aquaporins, ion channel proteins, and ABC transporters), terms related to pyruvate metabolism and oxidation‐reduction process, glutathione metabolism, and other glycan degradation (File S1). Moreover, of the genes enriched in transport‐related terms, more than one fifth were members of the SLC superfamily.

Genes in the species‐correlated turquoise module derived from WGCNA were enriched in 64 GO terms and eight KEGG pathways, including terms related to pyruvate metabolism, oxidoreductase activity, glutathione metabolism, transmembrane transport, FAA metabolism (such as tryptophan), response to stimulus, cytochrome P450, and DNA repair (File S1, *p* < 0.05). Besides, among the genes in another species‐correlated red module, FAA metabolism (including taurine, hypotaurine, alanine, aspartate, and glutamate), transport, succinate‐semialdehyde dehydrogenase [NAD(P)+] activity, glycogen metabolic process, oxidoreductase activity, transcription factor activity, and vitamin B6 metabolism were in the list of 26 GO terms and six KEGG pathways that were enriched (File S1, *p* < 0.05).

**FIGURE 7:**
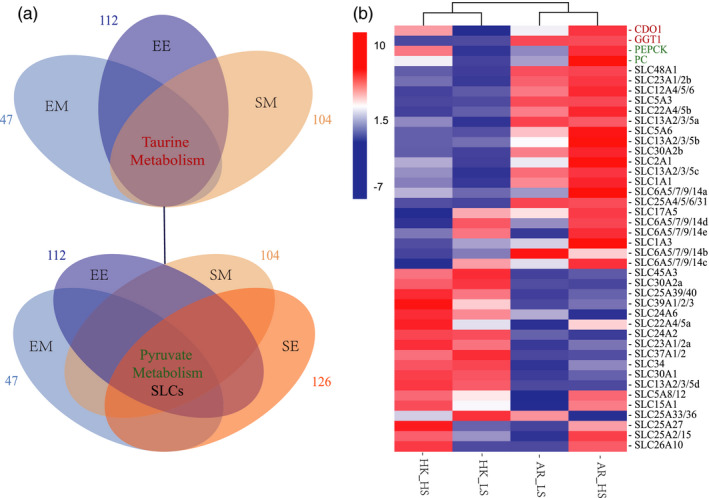
Candidate pathways and expression heatmap responding to both species divergence and salinity gradients. (a) The ellipses indicate enriched pathways in differentially expressed gene (DEG) sets and gene sets from weighted gene correlation network analysis (WGCNA). Taurine metabolism pathway (indicated on the core cross regions) was multiply enriched in genes in salinity (environmental)‐correlated modules (EM, light blue ellipse), in DEGs for environment effect (different expression between the salinity gradients, EE, dark blue ellipse), and in genes in species‐correlated modules (SM, light orange ellipse). Pyruvate metabolism and SLCs (indicated on the core cross regions) were multiply enriched in EM, EN, SM, and DEGs for species effect (different expression between the species, SE, dark orange ellipse). Other enriched pathways in each gene set were hidden. The numbers next to each ellipse indicate the total number of enriched pathways in each gene sets. 47 for EM: 39 GO and 8 KEGG enrichments in salinity‐correlated module; 112 for EE: 45 GO and 5 KEGG enrichments in salinity‐responsive DEGs for *Crassostrea ariakensis*, plus 54 GO and 8 KEGG enrichments in salinity‐responsive DEGs for *C. hongkongensis*; 104 for SM: 64 GO and 8 KEGG enrichments in species‐correlated turquoise module, plus 26 GO and 6 KEGG enrichments in species‐correlated red module; and 126 for SE: 62 GO and 5 KEGG enrichments in species divergent DEGs under high‐salinity conditions, plus 52 GO and 7 KEGG enrichments in species divergent DEGs under low‐salinity conditions. (b) Expression heatmap of genes contributed to the enrichment of the three pathways above. Red boxes indicate high expression, and blue boxes indicate low expression. Gene annotations are indicated on the right, among which the enriched genes in red (CDO1: cysteine dioxygenase 1, GGT1: gamma‐glutamyl transpeptidase 1) belong to the taurine metabolism pathway; enriched genes in green (PEPCK: phosphoenolpyruvate carboxykinase, PC: pyruvate carboxylase) belong to the pyruvate metabolism pathway; and other enriched genes in black are members of SLCs. AR: *C. ariakensis*; HK: *C. hongkongensis*; HS, high salinity; LS, low salinity

Several pathways enriched in DEGs between the two species and the species‐correlated WGCNA modules, such as taurine metabolism, pyruvate metabolism, and transporters (especially SLCs), were also enriched in environment‐responsive DEG sets and the salinity‐correlated WGCNA module (Figure 7). The 42 enriched genes in taurine metabolism, pyruvate metabolism, and SLCs exhibited divergent expression between the two oyster species with clustering in two groups according to species (Figure 7). All the enriched genes in taurine and pyruvate metabolism pathways showed high expression in *C. ariakensis* but not in *C. hongkongensis*, and most exhibited increased expression in the high‐salinity environment compared with those in the low‐salinity environment (Figure 7). As for enriched SLCs, approximately half of the 38 SLCs were upregulated in *C. ariakensis* in contrast to *C. hongkongensis*, and several among them, such as SLC1A3, SLC2A1, SLC5A6, and SLC6A5/7/9/14a, were highly expressed in the high‐salinity environment compared with those in the low‐salinity environment for *C. ariakensis*. The other half of the enriched SLCs were upregulated in *C. hongkongensis* in contrast to *C. ariakensis*, among which SLC25A33/36, SLC37A1/2, and SLC45A3 exhibited high expression in the low‐salinity environment compared with those in the high‐salinity environment for *C. hongkongensis* (Figure 7).

## 4 DISCUSSION

### 4.1 Divergences in salinity adaptation between *C. ariakensis* and *C. hongkongensis*

The culture experiment conducted in this study indicated phenotypic divergences caused by salinity adaptation between *C. ariakensis* and *C. hongkongensis* based on growth, survival, and physiological measurements. Although these species are partly sympatric in estuaries, they exhibited different fitness to salinity gradients. For the growth phenotype, the greater growth rate and percent survival of *C. ariakensis* in the high‐salinity environment suggested that it had higher fitness under high‐salinity conditions than under low‐salinity conditions. Conversely, *C. hongkongensis* had higher fitness to live under low‐salinity conditions than under high‐salinity conditions. Regarding the physiological responses to salinity change, we predominately measured the activity of enzymes related to respiratory metabolism and antioxidant processes. A lower MDA content observed for *C. ariakensis* in the high‐salinity environment compared with that in the low‐salinity environment suggested less oxidative damage, indicating the higher fitness of this species under high‐salinity conditions than under low‐salinity conditions. Although no significant difference in SOD activity was observed between the salinity gradients for *C. ariakensis*, the decreased MDA recorded in the high‐salinity environment compared with that in the low‐salinity environment may be attributed to the action of other antioxidant enzymes. The ratio of PK to PEPCK represents the transformation from moderate aerobic to challenged anaerobic metabolism in the thermal adaptation in other bivalves (Anestis et al., 2010; Ghaffari et al., 2019; Robert et al., 1995; Sokolova et al., 2012). Decreased PK:PEPCK ratio in *C. hongkongensis* under high‐salinity conditions compared with that under low‐salinity conditions suggested the activation of the anaerobic metabolism at high salinity, while *C. ariakensis* showed more anaerobic metabolism under low‐salinity conditions. Our findings indicate that *C. hongkongensis* had higher fitness under lower salinity conditions, whereas *C. ariakensis* had higher fitness under higher salinity conditions. Unlike previous studies on *C. ariakensis* and *C. hongkongensis* that demonstrated their similar optimal salinity range (10–25 ppt) (Wang et al., 2004; Xiao et al., 2018), our results further revealed the phenotypic divergences caused by salinity adaptation between the two oyster species to finer‐scale gradients within this salinity range (HS: 21.0 ± 1.7‰; LS: 13.6 ± 1.6‰). Our findings are consistent with a previous study, which reported that *C. hongkongensis* can thrive at salinity as low as 5 ppt, with high hyposalinity adaptability (Xiao et al., 2018).

### 4.2 Role of species effect and environment effect in the adaptive divergence

Consistent with the phenotypic divergences caused by salinity adaptation between the two species, PCA confirmed large gene expression variances (33.5%) of the total transcriptional variance demonstrated by PC1, which represents interspecies divergence. Intraspecies variation in gene expression plasticity in response to salinity gradients only occupied a small portion as PC2 accounted for 9.2% of the differential expression separating the high‐salinity environment from the low‐salinity environment for both species. In addition to our PCA result from a population treated with different salinities, the divergent transcriptional response between populations from different salinity gradients within *C. hongkongensis* was also supported by previous findings (Xiao et al., 2018). The intraspecies divergence related to salinity adaptation in *C. ariakensis* was supported by previous studies on the transcriptional response to environmental gradients (Liu et al., 2019) or on the population structure and genomic basis for salinity adaptation (Li et al., 2020, 2021; Wu et al., 2021) among populations from different habitats.

The SNP phylogenetic tree showed that oysters of the same species under the two salinity conditions mixed into one branch, indicating no significant genetic differentiation from the cultivation in different salinities in this study. Therefore, the major factor that caused the divergence in expression might be interspecies divergence rather than environment effect because the short‐term cultivation in changing salinity could not lead to genetic differentiation. In addition, the number of genes differentially expressed between the species and the salinity gradients demonstrated that more than 75% of the DEGs showed the species effect, suggesting that the differences in response to salinity were predominately mediated by interspecific adaptive differentiation. Consistently, the results of the WGCNA indicated that more than 70% of the genes in all modules were related to interspecies differences.

Our results suggest that when faced with changing environment conditions, such as salinity stress, species differences may have a larger effect than the changing environmental factors themselves, which has also been observed in studies on the reactions of organisms subjected to fire (Latutrie et al., 2019) and chemicals (Avci & Yaman, 2014). Our observations between species were reasonable in contrast to the transcriptomic variation of populations within *Crassostrea virginica*, where two reef‐source populations showed a greater environment effect than genetic effect on gene expression differences in response to different salinity environments (Eierman & Hare, 2016). Furthermore, transporters (especially SLCs), taurine metabolism pathway, and pyruvate metabolism pathway enriched in the salinity‐correlated module were in response to salinity gradients, which were supported by those of previous studies (SLCs: Boyle et al., 2015; Choi et al., 2003; Hoglund et al., 2011; Hui et al., 2014; Jiang et al., 2020; Kurita et al., 2008; Li et al., 2021; Menchini & Chaudhry, 2019; Nakajima et al., 2013; Wang et al., 2015; Wang et al., 2020; Wu et al., 2021; Zhou et al., 2018; Taurine: Meng et al., 2013; Wu et al., 2021; Pyruvate: Derakhshani et al., 2020; Gullian et al., 2018; Wang, Li, et al., 2017). These salinity‐responsive pathways also exhibited divergent responses between two oyster species for their enrichment of DEG sets for the species effect and in species‐correlated modules, emphasizing the role of these mechanisms in the formation of divergence in salinity adaptation between the two species. Moreover, the high expression for enriched genes in pyruvate and taurine metabolism pathway and SLCs for *C. ariakensis* may contribute to its hyperosmotic adaptation given their increased expression in the high‐salinity environment compared with that in the low‐salinity environment. Furthermore, the members of SLCs that showed increased expression for *C. hongkongensis* compared with *C. ariakensis*, especially those highly expressed under low‐salinity conditions compared with high‐salinity conditions, may also be responsible for the adaptation to low salinity of *C. hongkongensis*. These findings may underline the adaptive mechanisms for tolerating high or low salinity in marine mollusks.

### 4.3 Implications for oyster conservation and aquaculture

Oysters are widely distributed around the world and provide important ecological services as reef‐builders and filter‐feeders in estuaries and costal oceans, which are experiencing wide fluctuations in salinity. Oysters are also of economic importance because the aquaculture production of oysters amounts to 6.1 million metric tons with more than 85.3% produced in China (Fisheries & Aquaculture Department, 2021).

Given the increased salinity differences among estuaries (Cheng et al., 2020; Chou et al., 2013) and stress expected from low‐salinity extremes as the storm frequency and magnitude increases, our findings provide insights into fisheries management referring to species conservation in suitably salty environments and for the assessment of adaptive potential under rapid climate change. *C. hongkongensis* may have high adaptive potential in the face of decreasing salinity in South China (Cheng et al., 2020), while the sympatric *C. ariakensis* may has low fitness under such conditions. However, allopatric counterparts of *C. ariakensis* in North China may has a high capacity to adapt to estuaries with increasing salinity (Cheng et al., 2020; Chou et al., 2013), based on its higher fitness in high‐salinity environment observed in this study.

*Crassostrea hongkongensis*, one of the major commercial farming species in southern China, has suffered from mass mortality potentially correlated to high‐salinity disturbance, which increases the demand for farming of the sympatric *C. ariakensis*. The higher fitness of *C. hongkongensis* under low‐salinity conditions and of *C. ariakensis* under high‐salinity conditions indicates their adaptation to low‐ and high‐salt environments, respectively. These results provide theoretical support for the shift from *C. hongkongensis* to *C. ariakensis* in the aquaculture industry in some regions or times of year in South China with high salinity.

Our findings may guide the acclimatization culture to increase the adaptation to high salinity for *C. hongkongensis* and low salinity for *C. ariakensis* in oyster industry. Hybridization has long been considered an important source of genetic variation that leads to the adaptive evolution of novel phenotypes in new environments (Lewontin & Birch, 1966; Taylor et al., 2015). Hybridization with high‐salt‐adapted *C. ariakensis* has been utilized for the genetic modification of *C. hongkongensis* to improve their resistance to high salinity (Qin et al., 2020, 2021). Moreover, candidate genes, such as members of the SLC family, could be used for molecular assistant selection breeding for salinity‐resistant strains.

## 5 CONCLUSION

In this study, we conducted a novel comparative study on the adaptive divergence of two oyster species, *C. ariakensis* and *C. hongkongensis*, across naturally formed salinity gradients. Divergent phenotypes and transcriptomic expression profiles were observed between the two species in fine‐scale salinity gradients. *C. ariakensis* showed a higher fitness under high‐salinity conditions, and *C. hongkongensis* showed a higher fitness under low‐salinity conditions. Furthermore, the divergence in phenotypic and transcriptional responses was predominately due to the species effect between *C. ariakensis* and *C. hongkongensis*. Salinity‐responsive pathways, such as taurine metabolism, pyruvate metabolism, and SLCs, are responsible for the formation of the divergence in salinity adaptation between the two species, and these three pathways may be responsible for the divergence in salinity adaptation in mollusks. The pyruvate and taurine metabolism pathway and several SLCs may contribute to the hyperosmotic adaptation of *C. ariakensis*, and some SLCs may contribute to the hypoosmotic adaptation of *C. hongkongensis*. Our study provides novel insights into the mechanisms underlying salinity adaptation in marine mollusks, which will not only lay a theoretical foundation for predicting the adaptive potential of these species to climate change but also aid in resolving the mass mortality of *C. hongkongensis* in the aquaculture industry and conserving the germplasm resources of the two oyster species.

## CONFLICT OF INTEREST

There are no conflicts of interest to declare.

## Supporting information

Figure S1

Figure S2

Figure S3

Figure S4

Table S1

Table S2

Table S3

Table S4

File S1

File S2

Supplementary Material

Supplementary Material

## Data Availability

The transcriptome data for this study are available at the Sequence Read Archive (SRA) database under the accession number PRJNA810022.
