# Supplementary material for: Adaptive divergence and underlying mechanisms in response to salinity gradients between two Crassostrea oysters revealed by phenotypic and transcriptomic analyses
Source: Evol Appl. 2022 Apr 18;16(2):234–49. doi: 10.1111/eva.13370 (PMC9923467; doi:10.1111/eva.13370)
Supplement: Supplementary file 5 — Table S1 [file EVA-16-234-s005.docx]

| **subject** | **test name** | **groups** | **test statistic** | **DF** | **P value** |
| --- | --- | --- | --- | --- | --- |
| **salinity (Fig. 1b)** | Welch’s t-test | HS area vs. LS area | t = 115.0 | df = 2544 | < 0.0001 |
| **temperature (Suppl. Fig. 1)** | Welch’s t-test | HS area vs. LS area | t=8.595 | df=2556 | < 0.0001 |
| **log2(fold change) (Fig.5e)** | Mann-Whitney U test | environment effect vs. species effect | U=331980724.0 | \ | < 0.0001 |
| **log2(fold change) (Fig.5e)** | Mann-Whitney U test | environment effect vs. species-environment interaction | U=330485088.5 | \ | < 0.0001 |
| **log2(fold change) (Suppl. Fig. 3a)** | t-test | AR vs. HK | t=2.887 | df=176 | 0.0044 |
| **log2(fold change) (Suppl. Fig. 3b)** | t-test | AR vs. HK | t=2.519 | df=3925 | 0.0118 |
