# Supplementary material for: Adaptive divergence and underlying mechanisms in response to salinity gradients between two Crassostrea oysters revealed by phenotypic and transcriptomic analyses
Source: Evol Appl. 2022 Apr 18;16(2):234–49. doi: 10.1111/eva.13370 (PMC9923467; doi:10.1111/eva.13370)
Supplement: Supplementary file 6 — Table S2 [file EVA-16-234-s011.docx]

| **subject** | **test name** | **ANOVA table** | | | | **post-hoc analysis** | |
| --- | --- | --- | --- | --- | --- | --- | --- |
|  |  |  | **DF** | **F (DFn, DFd)** | **P value** | **groups** | **P value** |
| **shell height (Suppl. Fig. 2, 2019.09)** | two-way ANOVA | Interaction | 1 | F (1, 637) = 0.7185 | P=0.3970 | AR HS vs. AR LS | 0.036 |
|  |  | Species | 1 | F (1, 637) = 1003 | P<0.0001 | AR HS vs. HK HS | <0.0001 |
|  |  | Environment | 1 | F (1, 637) = 8.658 | P=0.0034 | AR HS vs. HK LS | <0.0001 |
|  |  | Residual | 637 |  |  | AR LS vs. HK HS | <0.0001 |
|  |  |  |  |  |  | AR LS vs. HK LS | <0.0001 |
|  |  |  |  |  |  | HK HS vs. HK LS | 0.4554 |
| **shell height (Suppl. Fig. 2, 2019.11)** | two-way ANOVA | Interaction | 1 | F (1, 232) = 24.44 | P<0.0001 | AR HS vs. AR LS | 0.8251 |
|  |  | Species | 1 | F (1, 232) = 383.7 | P<0.0001 | AR HS vs. HK HS | <0.0001 |
|  |  | Environment | 1 | F (1, 232) = 15.33 | P=0.0001 | AR HS vs. HK LS | <0.0001 |
|  |  | Residual | 232 |  |  | AR LS vs. HK HS | <0.0001 |
|  |  |  |  |  |  | AR LS vs. HK LS | <0.0001 |
|  |  |  |  |  |  | HK HS vs. HK LS | <0.0001 |
| **percent survival (Fig. 2b)** | two-way ANOVA | Interaction | 1 | F (1, 8) = 28.68 | P=0.0007 | AR HS vs. AR LS | 0.0209 |
|  |  | Species | 1 | F (1, 8) = 6.786 | P=0.0314 | AR HS vs. HK HS | 0.2834 |
|  |  | Environment | 1 | F (1, 8) = 0.004244 | P=0.9497 | AR HS vs. HK LS | 0.3414 |
|  |  | Residual | 8 |  |  | HK HS vs. AR LS | 0.3046 |
|  |  |  |  |  |  | AR LS vs. HK LS | 0.0022 |
|  |  |  |  |  |  | HK HS vs. HK LS | 0.0237 |
| **SOD (Fig. 3a)** | two-way ANOVA | Interaction | 1 | F (1, 21) = 2.195 | P=0.1533 | AR HS vs. AR LS | 0.9117 |
|  |  | Species | 1 | F (1, 21) = 67.16 | P<0.0001 | AR HS vs. HK HS | 0.0007 |
|  |  | Environment | 1 | F (1, 21) = 0.2149 | P=0.6477 | AR HS vs. HK LS | <0.0001 |
|  |  | Residual | 21 |  |  | AR LS vs. HK HS | 0.0001 |
|  |  |  |  |  |  | AR LS vs. HK LS | <0.0001 |
|  |  |  |  |  |  | HK HS vs. HK LS | 0.4352 |
| **MDA (Fig. 3b)** | two-way ANOVA | Interaction | 1 | F (1, 22) = 8.064 | P=0.0095 | AR HS vs. AR LS | 0.0024 |
|  |  | Species | 1 | F (1, 22) = 593.9 | P<0.0001 | AR HS vs. HK HS | <0.0001 |
|  |  | Environment | 1 | F (1, 22) = 15.84 | P=0.0006 | AR HS vs. HK LS | <0.0001 |
|  |  | Residual | 22 |  |  | AR LS vs. HK HS | <0.0001 |
|  |  |  |  |  |  | AR LS vs. HK LS | <0.0001 |
|  |  |  |  |  |  | HK HS vs. HK LS | 0.7375 |
| **PK:PEPCK (Fig. 3c)** | two-way ANOVA | Interaction | 1 | F (1, 23) = 6.621 | P=0.0170 | AR HS vs. AR LS | 0.8552 |
|  |  | Species | 1 | F (1, 23) = 0.2966 | P=0.5912 | AR HS vs. HK HS | 0.1683 |
|  |  | Environment | 1 | F (1, 23) = 1.808 | P=0.1919 | AR HS vs. HK LS | 0.9454 |
|  |  | Residual | 23 |  |  | AR LS vs. HK HS | 0.5278 |
|  |  |  |  |  |  | AR LS vs. HK LS | 0.4681 |
|  |  |  |  |  |  | HK HS vs. HK LS | 0.0255 |
| **CS (Fig. 3d)** | two-way ANOVA | Interaction | 1 | F (1, 28) = 2.882 | P=0.1007 | AR HS vs. AR LS | 0.9427 |
|  |  | Species | 1 | F (1, 28) = 123.6 | P<0.0001 | AR HS vs. HK HS | <0.0001 |
|  |  | Environment | 1 | F (1, 28) = 5.933 | P=0.0215 | AR HS vs. HK LS | <0.0001 |
|  |  | Residual | 28 |  |  | AR LS vs. HK HS | <0.0001 |
|  |  |  |  |  |  | AR LS vs. HK LS | <0.0001 |
|  |  |  |  |  |  | HK HS vs. HK LS | 0.0486 |
