# Supplementary material for: Adaptive divergence and underlying mechanisms in response to salinity gradients between two Crassostrea oysters revealed by phenotypic and transcriptomic analyses
Source: Evol Appl. 2022 Apr 18;16(2):234–49. doi: 10.1111/eva.13370 (PMC9923467; doi:10.1111/eva.13370)
Supplement: Supplementary file 7 — Table S3 [file EVA-16-234-s007.docx]

| Species | Salinity | Sample ID | Clean reads | Clean bases | GC Content | %≥Q30 | Total Reads | Mapped Reads |
| --- | --- | --- | --- | --- | --- | --- | --- | --- |
| *C. ariakensis* | High Salinity | F-1 | 20,571,345 | 6,163,787,194 | 43.09% | 95.10% | 41,142,690 | 32,496,544 (78.98%) |
|  |  | F-2 | 21,432,016 | 6,419,133,508 | 43.15% | 95.81% | 42,864,032 | 34,038,847 (79.41%) |
|  |  | F-3 | 24,338,586 | 7,292,004,312 | 43.20% | 95.88% | 48,677,172 | 38,779,011 (79.67%) |
|  |  | F-4 | 21,834,002 | 6,540,971,184 | 43.04% | 95.93% | 43,668,004 | 34,720,801 (79.51%) |
|  |  | F-5 | 20,742,630 | 6,215,765,990 | 42.91% | 95.78% | 41,485,260 | 32,986,440 (79.51%) |
|  | Low Salinity | H-1 | 22,039,909 | 6,603,926,520 | 43.35% | 95.02% | 44,079,818 | 34,770,558 (78.88%) |
|  |  | H-2 | 19,700,499 | 5,903,637,758 | 43.00% | 95.38% | 39,400,998 | 31,128,939 (79.01%) |
|  |  | H-3 | 19,199,575 | 5,754,180,284 | 43.14% | 96.00% | 38,399,150 | 30,380,731 (79.12%) |
|  |  | H-4 | 19,806,269 | 5,935,253,248 | 43.49% | 95.67% | 39,612,538 | 31,689,086 (80.00%) |
|  |  | H-5 | 20,847,103 | 6,245,167,446 | 43.34% | 95.60% | 41,694,206 | 33,111,941 (79.42%) |
| *C. hongkongensis* | High Salinity | J-1 | 31,141,217 | 9,299,012,682 | 41.87% | 93.79% | 49,393,076 | 40,627,390 (82.25%) |
|  |  | J-2 | 24,696,538 | 7,378,749,758 | 42.28% | 94.18% | 68,754,936 | 57,220,588 (83.22%) |
|  |  | J-3 | 34,377,468 | 10,241,140,186 | 43.53% | 93.87% | 53,264,680 | 43,133,866 (80.98%) |
|  |  | J-4 | 26,632,340 | 7,948,537,402 | 42.08% | 93.86% | 56,671,318 | 46,441,203 (81.95%) |
|  |  | J-5 | 28,335,659 | 8,463,967,264 | 42.67% | 93.80% | 51,886,048 | 42,535,651 (81.98%) |
|  | Low Salinity | K-1 | 25,943,024 | 7,771,020,996 | 42.43% | 94.03% | 55,622,984 | 45,631,273 (82.04%) |
|  |  | K-2 | 27,811,492 | 8,332,879,340 | 42.64% | 94.37% | 54,680,086 | 44,795,065 (81.92%) |
|  |  | K-3 | 27,340,043 | 8,168,341,750 | 42.62% | 94.13% | 62,024,928 | 50,870,708 (82.02%) |
|  |  | K-4 | 31,012,464 | 9,277,467,988 | 42.58% | 94.76% | 72,293,128 | 58,456,710 (80.86%) |
|  |  | K-5 | 36,146,564 | 10,820,344,030 | 42.08% | 94.37% | 72,293,128 | 58,456,710 (80.86%) |
