# Supplementary material for: Adaptive divergence and underlying mechanisms in response to salinity gradients between two Crassostrea oysters revealed by phenotypic and transcriptomic analyses
Source: Evol Appl. 2022 Apr 18;16(2):234–49. doi: 10.1111/eva.13370 (PMC9923467; doi:10.1111/eva.13370)
Supplement: Supplementary file 8 — Table S4 [file EVA-16-234-s012.docx]

|  | DEG Set | Reference Group | DEG Number | up-regulated | down-regulated |
| --- | --- | --- | --- | --- | --- |
| environment effect | AR HS vs. AR LS | AR HS | 101 | 37 | 64 |
|  | HK HS vs. HK LS | HK HS | 134 | 61 | 73 |
| species effect | AR HS vs. HK HS | AR HS | 1,538 | 867 | 671 |
|  | AR LS vs. HK LS | AR LS | 1,469 | 846 | 623 |
| species-environment interaction | AR HS vs. HK LS | AR HS | 1,640 | 939 | 701 |
|  | AR LS vs. HK HS | AR LS | 1,571 | 947 | 624 |
