# Supplementary material for: Adaptive divergence and underlying mechanisms in response to salinity gradients between two Crassostrea oysters revealed by phenotypic and transcriptomic analyses
Source: Evol Appl. 2022 Apr 18;16(2):234–49. doi: 10.1111/eva.13370 (PMC9923467; doi:10.1111/eva.13370)
Supplement: Supplementary file 12 — Supplementary Material [file EVA-16-234-s001.docx]

**Supplementary Figure 1.** Seawater temperature of sampling locations. Seawater temperature of the two sampling locations of the cultured *Crassostrea. ariakensis* and *C. hongkongensis* in high- or low-salinity environments, respectively. It was recorded during two months of acclimatization (September 2019 to November 2019) by a conductivity logger at 1 h intervals. Asterisks indicate significant difference (*****p* < 0.0001). Error bars indicate SD. The mean temperature in each site was indicated above the bars.

**Supplementary Figure 2.** Shell heights of *Crassostrea ariakensis* and *C. hongkongensis* acclimatized to two salinity gradients. Shell heights of the two species acclimatized at two sites with high- and low-salinity environments were separately measured at the beginning (September 2019) and the end (November 2019) of the acclimatization. Asterisks indicate significant differences (*****p* < 0.0001). Error bars indicate SD.

**Supplementary Figure 3.** Boxplots of absolute expression changes, |log_2_(Fold Change)|, between two salinity gradients for *Crassostrea ariakensis* (blue) and *C. hongkongensis* (yellow) in the differential expression genes showing (a)environment effect and (b) species effect. The boxes represent the distribution of each data sets. The five horizontal lines of each box represent the positions of the maximum, third quartile (Q3), median (Q2), first quartile (Q1), and minimum, respectively. “X” of each boxplot indicates the mean of the |log_2_(Fold Change)| for each gene set, which was 1.18 for *C. ariakensis*, 1.26 for *C. hongkongensis* in DEGs for environment effect (a), and 0.57 for *C. ariakensis*, 0.66 for *C. hongkongensis* in DEGs for species effect (b). The SD of each gene set was 0.14 for *C. ariakensis*, 0.22 for *C. hongkongensis* in DEGs for environment effect (a), and 1.00 for *C. ariakensis*, 1.26 for *C. hongkongensis* in DEGs for species effect(b). Asterisks indicate significant differences (**p* < 0.05, ** *p <* 0.01).

**Supplementary Figure 4.** Hierarchical cluster tree displayed expression correlation modules derived from WGCNA based on transcriptomic expression of all the orthologous genes in *Crassostrea ariakensis* and *C. hongkongensis*. Each leaf in the tree represents one orthologous gene of the two species. The major tree branches constituted eight modules labeled with different colors.

**Supplementary Table 1.** Details of statistic tests for the comparisons of environmental conditions between high- and low-salinity culture sites, and of |log_2_(Fold Change)| in gene sets showing environment effect, species effect, and species-environment interaction. DF: degree of freedom; AR: *Crassostrea ariakensis*; HK: *C. hongkongensis*; LS: low salinity; HS: high salinity.

**Supplementary Table 2.** Details of statistic tests for the comparisons of shell height, percent survival, and physiological indexes of *Crassostrea ariakensis* and *C. hongkongensis* acclimatized to high- and low-salinity environments for two months. The post-hoc analysis was Tukey's multiple comparisons test. DF: degree of freedom; AR: *C. ariakensis*; HK: *C. hongkongensis*; LS: low salinity; HS: high salinity.

**Supplementary Table 3.** Transcriptomic sequencing and assembly statistics for samples of *Crassostrea ariakensis* and *C. hongkongensis* acclimatized to high- and low-salinity environments for two months (n = 20), with the reference genomes of *C. ariakensis* and *C. hongkongensis* for the corresponding species, respectively.

**Supplementary Table 4.** Numbers of differential expression genes (DEGs) showing species effect, environment effect, and species-environment interaction through the comparison of each pair of experimental groups (AR_HS vs. HK_HS, AR_LS vs. HK_LS, AR_HS vs. AR_LS, HK_HS vs. HK_LS, AR_HS vs. HK_LS and AR_LS vs. HK_HS) based on transcriptomic expression. The numbers of upregulated and downregulated DEGs in contrast to corresponding reference group are also indicated in the table. AR: *C. ariakensis*; HK: *C. hongkongensis*; LS: low salinity; HS: high salinity.

**Supplementary File 1.** Ranked significant results (*p* < 0.05) of GO and KEGG functional enrichment analyses in differential expression genes (DEGs) showing environment effect (DEGs between high- and low-salinity gradients) and species effect (DEGs between *Crassostrea ariakensis* and *C. hongkongensis*), as well as genes included in species-correlated turquoise module, salinity-correlated green module, and species-correlated red module from WGCNA.

**Supplementary File 2.** Detailed information of the enriched differential expression genes between high- and low-salinity gradients and between *Crassostrea ariakensis* and *C. hongkongensis*, including GeneID, Length, count, log_2_FoldChange, Up/Down-Regulation, q value, annotation according to GO, KEGG, and Nr database.
